# Supplementary material for: Evaluating the operational efficiency of NBA teams on franchise value: An assessment of data envelopment analysis
Source: PLoS One. 2024 Mar 8;19(3):e0297797. doi: 10.1371/journal.pone.0297797 (PMC10923485; doi:10.1371/journal.pone.0297797)
Supplement: Attachment — Submitted filename: Review Comments to the Author and response.docx [file pone.0297797.s001.docx]

**Review Comments to the Author**

Please use the space provided to explain your answers to the questions above. You may also include additional comments for the author, including concerns about dual publication, research ethics, or publication ethics. (Please upload your review as an attachment if it exceeds 20,000 characters)

**Revier 1.**

| **Order** | **Comments** | **Response** |
| --- | --- | --- |
| 1 | Check the Abstract. The authors should be careful when writing the manuscript and formatting the Heading. | I checked and corrected some mistakes. |
| 2 | The authors mention 180 NBA teams. Are you doing a duplicate count? The NBA has 30 teams. | I understand that the writing was confusing. I revised the sentences to convey the idea of 30 NBA teams over 6 seasons (n=30, or 30 decision-making units). |
| 3 | Following up on the above point, even if you consider each team per season as unique, 29 out of 180 is not significant at all. The authors should articulate their work clearly. | Out of 180 teams, 29 (16%) may appear insignificant at first glance. However, it is essential to consider the unique features of professional leagues. In the NBA, 16 out of 30 teams compete in the postseason, and participating in the postseason holds special significance for professional sports players and teams. Despite this, many teams that played in the postseason were not included in the lists. Notably, even teams that reached the semi-finals did not make the lists. In this study, teams exhibiting perfect efficiency are fewer than three in each season, demonstrating both good game and financial performance efficiently.  To elaborate on this point, I have incorporated the following sentences into the discussion: “The DEA results with 30 NBA teams from 6 seasons (n=180) show that only 29 out of 180 (16%) teams achieved perfect operating efficiency among the sample teams. When considering the NBA's situation where 16 teams compete in the postseason each season, it implies a significant observation that, arithmetically, only three teams can demonstrate perfect efficiency each season. In other words, taking into account both game performance and financial outcomes, achieving 100% efficiency is highly challenging.” |
| 4 | The Introduction section is lengthy and often repetitive. You should be clear in your objective. | I made it much shorter than before by removing abundant sentences |
| 5 | How effective is the measure of winning percentage? Winning two matches in 2 games is not the same as winning ten matches in 10 games, where both will have 100% wins. | I believe there was a misunderstanding at this point. All teams play an equal number of 82 games in a standard season. The winning percentage is a variable measured after the season ends. We contend that a single season in the NBA provides sufficient time to assess the overall capability of a team. Consequently, there is no difference in the meaning of winning percentage based on the stages of each season. |
| 6 | What is your dependent variable? The authors need to explain clearly. Is it an incremental value? If so, how are you measuring it? For the benefit of the readers, you should add a table explaining what each variable means. | The dependent variable is an incremental value per year based on Forbes' annual franchise valuations. Forbes calculates these values considering various aspects such as team performance, financial earnings, etc. I have attempted to convey this information in multiple ways throughout the manuscript. Additionally, we have included explanations of the variables used to perform Data Envelopment Analysis (DEA) in Table 2. |
| 7 | In Table 4 report the VIF values as well. Also, the increase in Adjusted-R2 seems to be much higher – too much higher values can occur due to multicollinearity as well. So, you should estimate the BIC values and the drop in BIC (See AE Raftery 1995 work https://www.jstor.org/stable/271063) | The VIFs of Contested Shots (33.37), Loose Balls Recovered (19.19), and Deflections (11.89) were excessively high, even though they were considered control variables. Consequently, we sequentially removed each of these three variables until all the VIFs fell below 5. The table presents the results of the VIF test with the remaining variables Variables \| Drive & pass % \| Secondary assists \| Potential assists \| Passes made \| Catch & shoot points \| Screen assists points \| Loose balls recovered \| Compensation \| Population Increase \| Operating Efficiency; VIF \| 1.40 \| 2.14 \| 2.21 \| 1.62 \| 2.44 \| 4.61 \| 3.92 \| 3.24 \| 1.22 \| 1.28 To explain this, we put these sentences: “We conducted VIF tests prior to the regression analysis, and three variables (contested shots, loose balls recovered, and deflections) were excluded due to their high VIF scores (33.37, 19.19, and 11.859, respectively). Other variables showed an acceptable VIF level ranging from 1.22 to 4.61” We re-conducted the regression analysis with revised control variables. Table 4 presents the results, and the corresponding explanation is as follows: “The dependent variable in the regression is the annual increase in team value, as determined by Forbes' annual franchise valuations. Table 4 presents the results. In Model 1, we included various control variables such as human assets, the budget of the year, and the population increase of the year. This was done because the dependent variable is the increased value of the team each year. Model 2 shows the role of operating efficiency in predicting incremental organizational value (B=462.21, p<.001) after controlling for human assets and budget. Furthermore, the model fit was significantly increased (delta R-square=0.15, p<.001; delta BIC=34.29, p<.001).” |

**Reviewer 2**

| **order** | **Comments** | **Response** |
| --- | --- | --- |
| 1 | While reading this paper, I could not stop thinking about some other variables that might be directly associated with franchise value and that are not mentioned by the authors such as location (coastal cities, large metropolises, small cities) and possibly the total number of titles in a franchise’s history. In this direction, I would like to read from the authors something that justifies the exclusion of such variables in the present analysis or if such variables can be thought to be already encompassed by correlated indicators already present in their work. | Your comments are very insightful, and we carefully considered them, leading us to re-analyze additional data. Subsequently, we have partially incorporated your suggestions.  Firstly, it's crucial to clarify that the dependent variable is not the Forbes franchise value itself but rather the increase in Forbes franchise value. This distinction is important as constant variables, such as location, may impact Forbes franchise value, but they might not directly contribute to the increase in Forbes franchise value.  To illustrate, consider the Golden State Warriors, who ranked 1st in NBA franchise value in 2023. However, their value was 28th out of 29 teams in 2004. Despite the location remaining constant, the franchise value increased from 28th to 1st as the Warriors evolved into one of the greatest teams in NBA history. Conversely, the Detroit Pistons held the 4th position in franchise value but dropped to 23rd in 2023. In the Pistons' case, while the location remained the same, the franchise experienced a shift from a winning culture after 2004 to a rebuilding phase in the 2020s.  We acknowledge that city conditions, especially factors like population size, may influence changes in Forbes franchise values. These changes can reflect fluctuations in potential market size and even the economic situation of the city.  To address this consideration, we have added population changes as a control variable. The following sentences are inserted to reflect this adjustment:  "Table 4 presents the results. In Model 1, we included various control variables such as human assets, the budget of the year, and the population increase of the year. This was done because the dependent variable is the increased value of the team each year. Changes in city conditions, such as population change, may influence the franchise value change in the city." |
| 2 | While discussing the importance of star players in a team, for instance, the authors do not mention any intrinsic geographical or historical characteristics of cities (franchises) that might help to attract star players or that may drive them away. | It was a particularly insightful and interesting area of investigation, prompting us to delve into the real story. However, the additional research conducted to incorporate your suggestion did not align with the hypothesized outcomes. Here's the narrative:  Between 2011 and 2023, there were approximately 1,577 free agent contracts in the NBA. We sampled the top 10 largest contracts each year, resulting in 130 contracts. From this subset, we focused on unrestricted free agents, amounting to 95 players. Notably, 56 unrestricted free agents (59%) chose to re-sign with their current teams. This trend is likely influenced by the NBA's Collective Bargaining Agreement (CBA), enabling current teams to offer longer and more lucrative contracts. Hence, we can assert that contract size stands out as one of the pivotal factors in attracting star players.  Conversely, 39 players opted to join a new team. Within this group, the only instance where geographical location or a big city played a significant role in attracting a star player was LeBron James, who signed with the Los Angeles Lakers in 2018. Players like Kevin Durant (2019), Kyrie Irving (2019), and Jimmy Butler (2019) departed from their teams due to locker room issues and a desire for new challenges. Others, including Jalen Brunson (2023), Dillon Brooks (2023), and Bruce Brown (2023), left their teams seeking larger roles and more substantial contracts. Additionally, players like Kevin Durant (2016) and Steve Nash (2012) signed with new teams primarily for championship aspirations. Therefore, our findings suggest that the primary motivations for star players moving to new teams are driven by financial considerations, seeking larger roles, or pursuing championship opportunities, rather than being primarily influenced by geographical locations or the historical characteristics of a city.  Additional Data Caveat:   - The population data is sourced from macrotrends.net. - We assumed that the Brooklyn Nets and New York Knicks share the same population. - We assumed that the Los Angeles Clippers and Los Angeles Lakers share the same population. - We assumed the population for the Utah Jazz is based on the city of Salt Lake City rather than the entire state of Utah. - We used the population of Toronto for the Toronto Raptors. However, it is important to note that Toronto has the potential to represent the entire population of Canada as they are the only NBA team in the country.   Although we attempted to elucidate these considerations, we found that they may not be fitting within the current context. Consequently, these sentences are not included in the text |
